# Combination of Fe/Cu –chelators and docosahexaenoic acid: an exploration for the treatment of colorectal cancer

**DOI:** 10.18632/oncotarget.17807

**Published:** 2017-05-11

**Authors:** Nanhui Yu, Hong Zhu, Yuan Yang, Yiming Tao, Fengbo Tan, Qian Pei, Yuan Zhou, Xiangping Song, Qiurong Tan, Haiping Pei

**Affiliations:** ^1^ Department of Gastrointestinal Surgery, Xiangya Hospital, Central South University, Changsha 410008, P.R. China; ^2^ Department of Pharmacy, Changsha Hospital for Maternal and Child Health Care, Changsha 410007, P.R. China; ^3^ Dong Medicine Key Laboratory of Hunan Province, Department of Laboratory medicine, Hunan University of Medicine, Hunan 418000, P.R. China

**Keywords:** combination of Fe/Cu –chelators and docosahexaenoic acid, myeloid cell leukemia-1 (Mcl-1), apoptosis, ubiquitination, colorectal cancer

## Abstract

Colorectal cancer (CRC) is one of the major causes of cancer deaths in the world. 5-fluorouracil (5-FU) -based chemotherapy is a common choice for patients with CRC; unfortunately, the benefit is rather limited due to the acquisition of drug resistance. Therefore, the alternative therapeutic strategies are required. The activation of autophagic mechanism was considered as the main cause of the acquisition of drug resistance in 5-FU treatment. Docosahexaenoic acid (DHA), a fatty acid, has been regarded as an efficient anticancer agent and can improve the drug resistance in conventional cancer therapy by a low basal level of autophagy in colon cancer cells. Moreover, removal of iron or copper by metal chelators could cause ROS levels increase and mediate cancer cell cytotoxicity led by autophagy. In the present study, we constructed a combination of 5-FU, 1:1 mixture of metal chelators di-2-pyridylketone 4-cyclohexyl-4-methyl-3-thiosemicarbazone hydrochloride (DpC) and N, N, N’, N’-tetrakis-[2-pyridylmethyl]-ethylenediamine (TPEN) named DTN, and DHA to evaluate the anticancer effect of this combination, compared to the traditional 5-FU-based chemotherapy; further we investigated the underlying mechanism. Through inducing ROS-mediated degradation of Mcl-1 ubiquitination, the triple combination of 5-FU, DTN and DHA resulted in the elevated apoptosis in CRC cells, thus to reduce the tumor size and weight. Taken together, this study suggests the triple combination of 5-FU+DTN+DHA exhibits an effective anticancer activity of overcoming drug resistance in colorectal cancer, mechanism as the elevated apoptosis mediated by an increase of ROS and Mcl-1 ubiquitination, may be a novel strategy for clinical colon cancer treatment.

## INTRODUCTION

Colorectal cancer, also known as large bowel cancer, can involve aberrant growths in the appendix, colon and rectum, which is considered to be one of the three most prevalent types of cancer in human [1, 2]. Despite the remarkable achievements in precautionary measures and diagnotic techniques and the improvements in chemotherapy, the median overall survival period of colorectal cancer patients with metastatic is only 24 months due to the acquisition of multidrug resistance or non-selective cytotoxicity, which represents a critical challenge in cancer therapy. Currently, 5-FU is the first line therapy for colorectal cancer [3]. However, the anticancer effect by 5-FU is often hampered by the development of drug resistance or non-selective cytotoxicity in the course of chemotherapy.

Autophagy is an evolutionarily conserved self- degradation process by which cells degrade and renew cellular molecules and organelles. During autophagy, parts of the cytoplasm and cellular organelles are engulfed within a double-membrane vesicle known as autophagosome. The autophagosomes then fuse with lysosomes and their contents are degraded by lysosomal proteases [4]. Autophagy is considered as a protective mechanism by which cells eliminate unwanted or damaged materials to prevent carcinogenesis. However, tumor cells can utilize autophagy to survive cellular stress, such as hypoxia, nutritional deficiency, and chemotherapy [5, 6]. Recent studies have shown that induction of autophagy facilitates cancer cells’ resistance to drug-induced apoptosis [7, 8]. The activation of autophagic mechanism was considered as the main cause of acquired resistance in chemotherapy, which leads to apoptosis tolerance through the cyclic utilization of intracellular degradation product [9].

Interestingly, Docosahexaenoic acid (DHA, the chemical structure was referred in supplement), has been revealed to have anticancer activity and improve the drug resistance effect of conventional cancer therapy by a low basal level of autophagy in SW620 cells [10]. Furthermore, DHA also exhibits the ability to activate cellular ubiquitin–proteasome system (UPS) and the pro-apoptotic effect on tumor cells, which were associated with the overproduction of mitochondrial reactive oxygen species (ROS) [11–13]. Mcl-1, a member of the well-known apoptosis-related Bcl-2 family, inhibits apoptosis by binding to the pro-apoptotic proteins such as Bax, Bak or Bim [14]. Previous studies showed a close association between Mcl-1 dysfunction and colorectal cancer; Mcl-1 ubiquitin degradation was considered as a key target of reversing the resistance of colorectal cancer [15, 16]. However, the underlying antineoplastic mechanism of UPS activation induced by DHA is yet unclear.

Besides drug resistance, non-selective cytotoxicity of anticancer agents remains a huge challenge. A number of studies revealed that chelators of transition metals such as iron and copper are likely to play important roles in the development and growth and neoplasms. Iron is an essential element necessary for a variety of crucial metabolic processes, including ribonucleotide reductase, which catalyzes the rate-limiting step in DNA synthesis [17]. Copper is also an essential micronutrient that detoxificated reactive oxygen species (ROS) via superoxide dismutase (SOD) and in the cross-linking of elastin and collagen [18, 19]. Moreover, copper is an essential co-factor for the formation of new blood vessels, a process termed angiogenesis [20]. Interestingly, previous studies showed that cancer cells are addicted to high iron levels and accumulate the metal through transferrin-dependent uptake [21, 22]; and the high levels of copper concentrated in cancer cells is presumed to be important in both angiogenesis and metastasis [23]. Since then, the targeted removal of iron or copper in cancer cells by metal chelators designed specifically for the treatment of cancer have been developed with the thiosemicarbazone, di-2-pyridylketone 4-cyclohexyl-4-methyl-3-thiosemicarbazone hydrochloride (DpC, the chemical structure was referred in supplement), and the acyclic amino metal chelator N, N, N’, N’-tetrakis-[2-pyridylmethyl]-ethylenediamine (TPEN, the chemical structure was referred in supplement). Mechanistically, thiosemicarbazones can bind to both iron and copper, leading to the formation of redox-active complexes that produce ROS which induces cancer cell cytotoxicity [24–27]. Further, thiosemicarbazone-induced lysosomal disruption has also been reported to lead to persistent synthesis of autophagosome which potentiates cell death [26]. TPEN has a remarkably high affinity for a broad spectrum of metal ions, including copper, iron and zinc and can induce meiotic arrest in oocytes [27]. Further, some studies showed that TPEN-mediated metal chelation results in selective killing of HCT116 colon cancer cells without affecting normal cells; TPEN blocks the increase in phospho-Erk and LC3-II levels, and attenuates cell death [28].

In view of the roles of omega-3 fatty acid and metal chelators, several studies showed these anticancer characteristics of overcoming drug resistance and selective cytotoxicity in cancer cells by the mechanisms of ROS involvement or regulation of cellular microenvironment. Here, a triple combination of 5-FU combined by metal chelators and omega-3 fatty acid related to targeting the chelation of metal and activation of UPS is proposed, which might provide more effective therapeutic agent in chemotherapy of colorectal cancer.

## RESULTS

### Effects of 5-FU, DTN and DHA on cell viability and ROS levels

**Figure 1:**
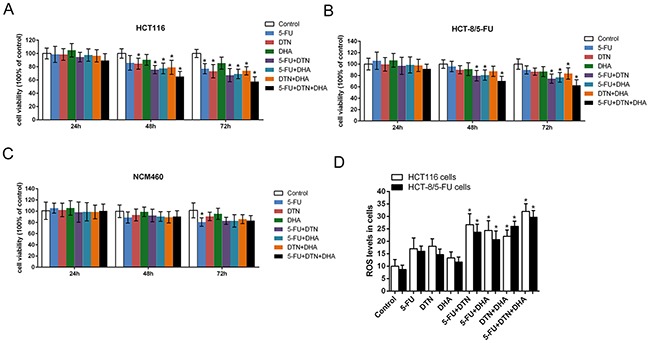
Characteristic of cell viability and ROS levels following different treatment in HCT116, HCT-8/5-FU or NCM460 cells **(A-C)** Characteristic of cell viability treated by single or combined treatment of 5-FU, DTN or DHA in HCT116 cells (A), HCT-8/5-FU cells (B) and NCM460 cells (C) were determined by MTT assay. **(D)** Characteristic of ROS levels following different treatment for 24 h in HCT116 and HCT-8/5-FU cells were determined by DCFH-DA assays. *P <0.05 showed a significant difference compared to control group.

Following single treatment of 5-FU (5 μg/mL), DTN (DpC: TPEN, 2.5 mM, respectively) or DHA (80 μM) for 24 h, 48 h and 72 h, result of MTT assay showed a time-dependent cytotoxicity in human colorectal cancer HCT116 cells, and two combined treatment of 5-FU+DTN, 5-FU+DHA and DTN+DHA showed a similar decrease of cell viability in HCT116 and HCT-8/5-FU cancer cells, while the change of cell viability was blunt in non-cancerous NCM460 cells at 48h. Interestingly, a triple combination of 5-FU+DTN+DHA resulted in a more noticeable decrease of cell viability in both HCT116 and HCT-8/5-FU cells (Figure 1A, 1B, 1C and Table 1), indicating a significantly higher sensitivity of cancerous cells as compared to their non-cancerous NCM460 cell counterpart. Following single treatment of 5-FU, DTN and DHA for 48 h, result of DCFH assay showed a slight up-regulation of cellular ROS levels compared to vehicle control in HCT116 and HCT-8/5-FU cells while the difference was not significant. The combined treatment of two drugs or three drugs showed the higher ROS levels than that of single treatments. Noticeably, the triple treatment showed the highest ROS levels among all treatments (Figure 1D). The result confirmed the reinforced cytotoxicity by the combined treatment of 5-FU, DTN and DHA on human colorectal cancer cells.

**Table 1:** Cells viability in response to different treatment in HCT116, HCT-8/5-FU or NCM460

Rate of cell survival (%)								
HCT116	Control	5-FU	DTN	DHA	5-FU+DTN	5-FU+DHA	DTN+DHA	5-FU+DTN+DHA
24h	100.03±8.08	98.61±12.04	98.49±8.79	104.80±10.12	94.65±7.14	97.62±9.24	96.51±9.22	89.51±10.28
48h	101.21±7.14	85.85±10.96	84.62±8.23*	90.43±8.08	75.71±6.16*	77.50±8.05*	79.14±10.56*	65.28±7.55*
72h	99.23±6.18	77.10±7.69*	73.24±10.02*	85.47±9.08	67.35±9.90*	69.14±7.11*	73.92±6.17*	57.80±7.13*
HCT-8/5-FU								
24h	103.1±10.33	105.84±15.12	99.50±11.82	106.57±12.05	96.90±15.01	98.55±14.09	97.59±10.74	91.66±8.09
48h	98.92±7.42	95.81±9.17	90.02±7.05	91.16±11.21	79.53±9.24*	80.70±9.12*	87.31±9.05	70.36±8.27*
72h	100.09±9.17	89.65±7.21	84.60±6.31	86.80±8.84	74.24±8.15*	76.54±8.56*	83.42±10.07*	62.73±9.78*
NCM460								
24h	100.78±15.41	105.40±8.87	102.20±12.02	105.7±13.01	98.22±18.06	98.93±16.23	98.57±12.04	100.34±12.23
48h	99.75±11.23	88.68±10.01	93.24±10.44	98.75±8.43	92.31±11.23	90.40±8.54	89.21±10.15	90.52±10.14
72h	101.43±13.32	81.56±7.55*	90.60±7.51	95.22±9.86	82.78±6.34	82.65±11.07	85.75±7.91	83.28±8.77

Notes: Data was showed as $\bar{X}\pm S_{d}$n = 5. * p<0.05 showed a significant difference compared to control group

### Effects of 5-FU, DTN, DHA on apoptosis and autophagy in cancer cells

**Figure 2:**
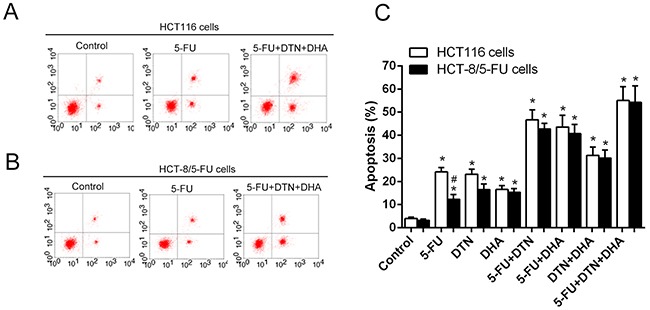
Characteristics of apoptosis in HCT 116 cells and HCT-8/5-FU cells following different treatment of 5-FU, DTN or DHA **(A)** and **(B)** After different treatment for 48h, Flow cytometry analysis of apoptosis in HCT116 cells (A) and HCT-8/5-FU cells (B) (part of the FACS data). **(C)** Dynamic trend of apoptosis in HCT116 or HCT-8/5-FU cells following different treatment of 5-FU, DTN or DHA. *P <0.05 showed a significant difference compared to control. # *P* <0.05 showed a significant difference between HCT116 cells and HCT-8/5-FU cells.

**Figure 3:**
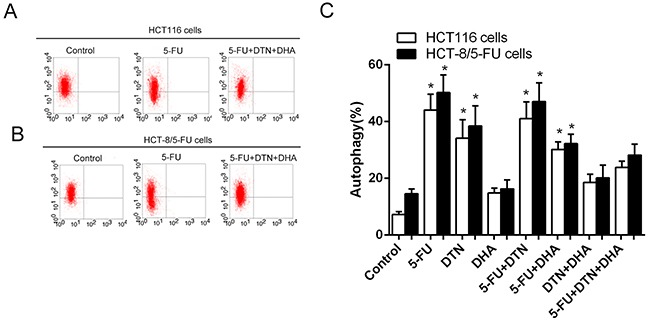
Characteristics of autophagy in HCT116 cells following different treatments of 5-FU, DTN and DHA **(A)** and **(B)** After different treatment for 48h, Flow cytometry analysis of autophagy in HCT116 cells (A) and HCT-8/5-FU cells (B) (part of the FACS data). **(C)** Dynamic trend of autophagy in HCT116 or HCT-8/5-FU cells following different treatment of 5-FU, DTN or DHA. *P <0.05 showed a significant difference compared to control group.

As shown in Table 2 and Figure 2, treatment of both HCT116 and HCT-8/5-FU cancer cells with combined 5-FU and DTN or DHA markedly an increased apoptosis compared to a single treatment of that, and the apoptotic change was similar in both cells except for 5-FU group showed the more resistant in HCT-8/5-FU cells. However, for the triple combination of 5-FU + DTN + DHA exposed to cells, the most significant apoptosis were revealed in both cells and the apoptotic difference in HCT116 and HCT-8/5-FU cells was not significant, indicating an overcome of drug resistance in HCT-8/5-FU cells. For the detection of autophagy assay, autophagy-mediated vacuole formations was observed in HCT116 and HCT-8/5-FU cells stained with AO following different treatments. Results showed that an elevated level of autophagic vacuole was observed in single or combination of 5-FU, DTN and DHA treatment group, compared to vehicle control, while the autophagic vacuole formation was at the highest level in the 5-FU group and at the lower level in treatment of DHA or combination of DHA plus DTN, indicating the inhibition of autophagy was associated with treatment of DHA. Interestingly, the lower level of autophagy accompanied by the highest apoptosis was observed in the combined treatment of 5-FU+DHA+DTN in both cells (Figure 3).

**Table 2:** Characteristic of apoptosis and autophagy in HCT116 cells and HCT-8/5-FU cells

Groups	HCT116 cells (%)		HCT-8/5-FU cells (%)	
	Apoptosis	Autophagy	Apoptosis	Autophagy
Control	4.01±0.98	7.21±1.64	3.15±0.95	14.52±2.88
5-FU	24.15±3.14*	44.02±9.70*	12.25±3.54^#^	50.15±10.75*
DTN	23.14±3.85*	34.12±11.25*	16.47±4.17*	38.41±12.33*
DHA	16.55±2.78*	14.76±3.08	15.34±2.74*	16.24±5.45
5-FU+DTN	46.62±7.46*	41.01±10.28*	42.68±4.37*	47.03±11.45*
5-FU+DHA	43.52±8.94*	30.14±4.66*	40.69±6.85*	32.15±5.88*
DTN+DHA	31.26±6.28*	18.54±4.95	30.14±5.95*	20.14±7.65
5-FU+DTN+DHA	55.02±10.44*	23.80±3.87	54.24±12.33*	28.15±6.67

Notes: Data was showed aspercent (%), n = 3, *P <0.05 showed a significant difference compared to control group. # *P* <0.05 showed a significant difference between HCT116 cells and HCT-8/5-FU cells.,

### Association of ROS levels and apoptosis- or autophagy-related proteins

**Figure 4:**
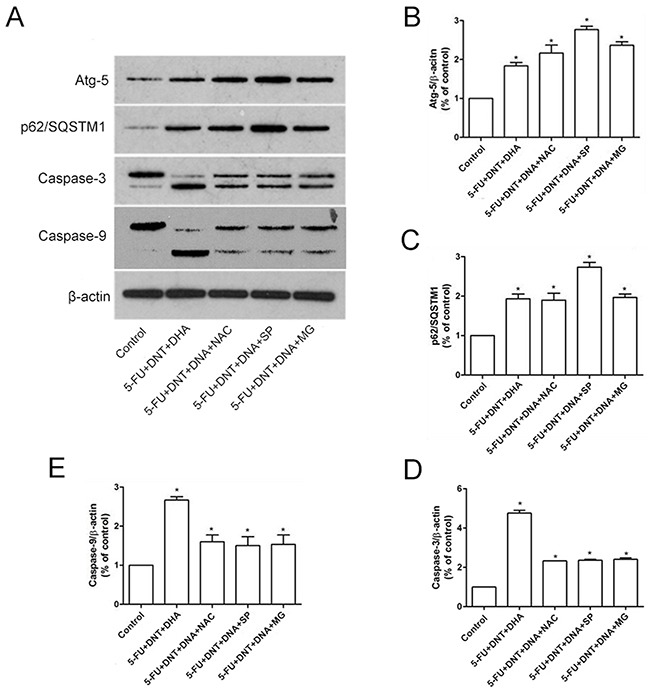
Western-Blot analysis of apoptosis- or autophagy- related proteins **(A)** The protein expression of Atg-5, p62/SQSTM1, Caspase-3 and Caspase-9 under different treatments were detected using western blotting. β-actin was used for loading control. **(B-E)** The densitometric analysis of Atg-5 (B), p62/SQSTM1 (C), Caspase-3 (D) and Caspase-9 (E) were exhibited in the right panels. *P <0.05 showed a significant difference compared to control.

As the Table 3 shown, the triple combination of 5-FU, DTN and DHA showed the higher elevation of ROS levels compared to vehicle control, and the elevated levels of Caspase-3 and Caspase-9 were also revealed, while the levels of autophagy-related Atg-5 or p62/SQSTM1 expression showed a slight up-regulation compared to control. Further, following cells were pretreated by antioxidant NAC to scavenge ROS before scheduled triple combination treatment, the increased levels of ROS and Caspase-3 or Caspase-9 were reversed in HCT116 cells, and the levels of Atg-5 or p62/SQSTM1 expression were still at elevated levels. However, following the interference of Spermidine (an autophagy inducer) or MG132 (a proteasome inhibitor), compared to triple combination of 5-FU+DTN+DHA, the levels of ROS showed a slight down-regulation, and the levels of Atg-5 or p62/SQSTM1 expression was still at a relatively high level, while the levels of Caspase-3 or Caspase-9 expression showed a significant down-regulation (Figure 4 and Table 3).

**Table 3:** Characteristic of ROS levels and apoptosis- or autophagy- related proteins

Group	Vehicle control	5-FU+DTN+ DHA	5-FU+DTN+ DHA +NAC	5-FU+DTN+ DHA+SP	5-FU+DTN+ DHA+MG
ROS levels	100.81± 4.43	178.3±24.19*	112.3±19.49^▲^	162.85±18.20*	198.15±24.45*
Atg-5	12.81 ± 2.11	26.31± 2.81*	48.07±3.79*^▲^	66.09±4.51*^▲^	41.75±5.43*^▲^
p62/SQSTM1	8.26 ± 1.24	22.30± 3.44*	36.23±3.79*^▲^	55.28±5.98*^▲^	33.44±4.93*^▲^
Caspase-3	7.04 ± 1.09	33.69± 6.65*	16.41±2.57*^▲^	16.61±1.98*^▲^	17.08±3.44*^▲^
Caspase-9	6.15± 1.21	29.18± 9.12*	15.08±2.43*^▲^	14.52±2.04*^▲^	14.31±1.33*^▲^
Ub-Mcl-1	6.23± 1.12	28.17± 5.08*	15.51± 3.17*^▲^	16.27± 3.31*^▲^	40.60± 6.49*^▲^

Notes: Caspase-3 indicates the cleaved 17 kD fragments of Caspase-3; Caspase-9 indicates the cleaved 35 kD fragments of Caspase-9, Ub-Mcl-1 indicates the level of Mcl-1 ubiquitination; Data was showed as X¯± Sd, n = 3,. *P <0.05 showed a significant difference compared to control, ^▲^*P* <0.05 showed a significant difference compared to treatment of 5-FU+DTN+DHA group.

**Figure 5:**
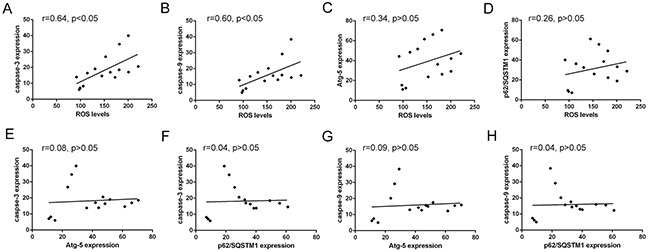
Correlated trend between ROS levels and Atg-5, p62/SQSTM1, caspase-3 or caspase-9 expression **(A)** and **(B)** Scatter diagram between ROS levels and apoptosis-related proteins caspase-3 and caspase-9 expression. **(C)** and **(D)** Scatter diagram between ROS levels and autophagy-related proteins Atg-5 and p62/SQSTM1 expression. **(E)** and **(F)** Scatter diagram between caspase-3 expression and autophagy-related proteins Atg-5 and p62/SQSTM1 expression. **(G)** and **(H)** Scatter diagram between caspase-9 expression and autophagy-related proteins Atg-5 and p62/SQSTM1 expression.

In addition, the correlation analysis (Figure 5 and Table 4) showed a positive correlation between ROS levels and Caspase-3, Caspase-9, while the correlation between ROS levels and the expression of autophagic Atg5 and p62/SQSTM1 was not significant. Also, the correlation between the levels of Atg5 or p62/SQSTM1 and Caspase-3, Caspase-9 was not significant (Figure 5A-5D). Also, the correlation between the levels of Atg5 or p62/SQSTM1 and Caspase-3 or Caspase-9 was no significant (Figure 5E-5H). The results indicated cellular ROS levels may promote the activation of apoptosis in HCT116 cells. For the not synchronous characteristic of ROS levels with the change of autophagy-related Atg5 or p62/SQSTM1 expression, the result indicated cellular ROS levels may not be the only factor regulated autophagy in HCT116 cells.

**Table 4:** Correlation between ROS levels and apoptosis- or autophagy- related protein or polyubiquitination of Mcl-1

	ROS levels	Atg-5	p62/SQSTM1	Caspase-3	Caspase-9	Ub-Mcl-1
ROS levels	1.00	0.34	0.26	0.64*	0.60*	0.86*
Atg-5	0.34	1.00	0.92*	0.08	0.09	0.03
p62/SQSTM1	0.26	0.92*	1.00	0.04	0.04	0.21
Caspase-3	0.64*	0.08	0.04	1.00	0.98*	0.53*
Caspase-9	0.60*	0.09	0.04	0.99*	1.00	0.47
Ub-Mcl-1	0.60*	0.19	0.12	0.57*	0.52*	1.00

Note: * *P* < 0.05 indicates a significant correlation coefficient.

### Association of Mcl-1 ubiquitination and apoptosis or autophagy

**Figure 6:**
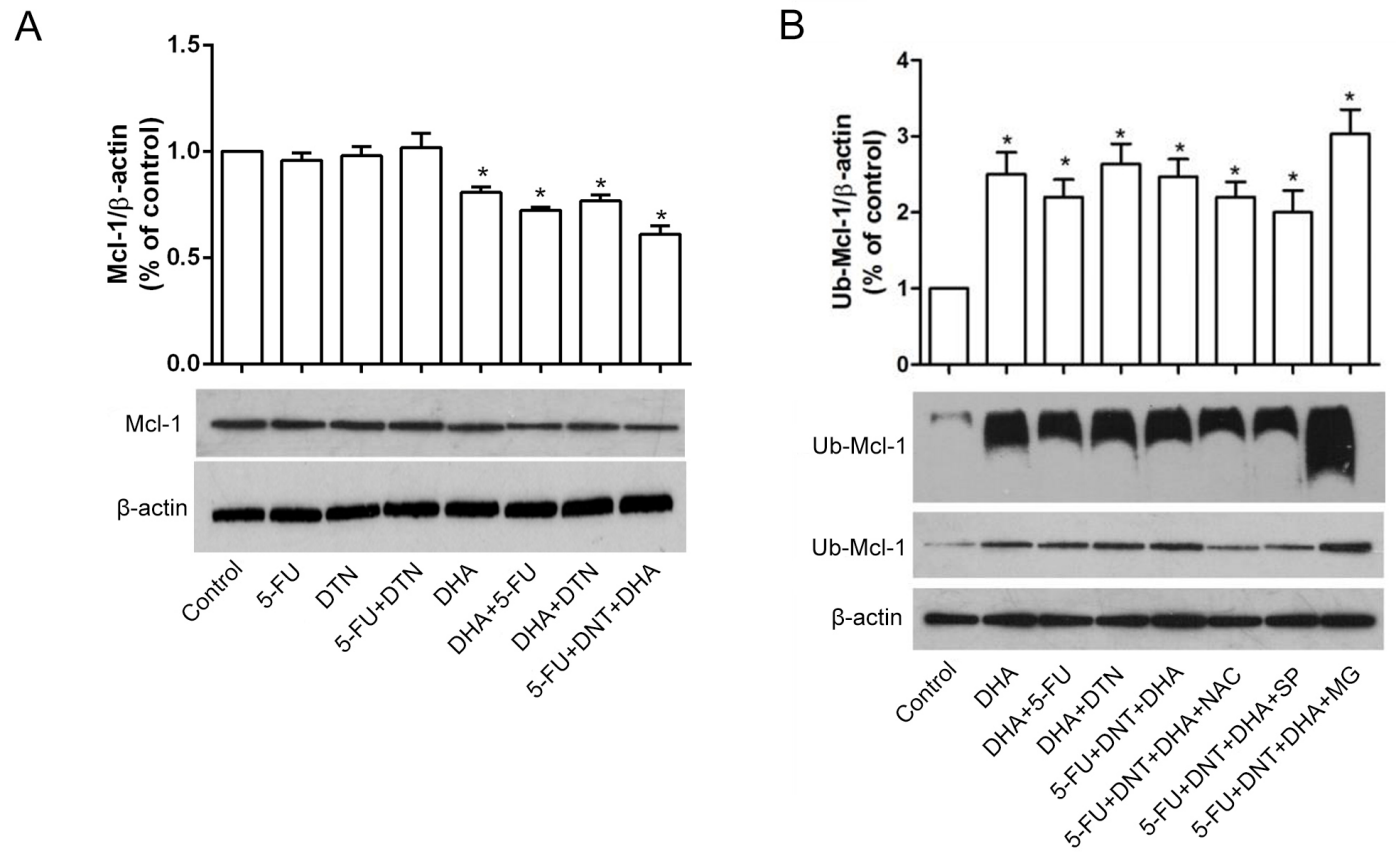
Characteristics of Mcl-1 expression and ployubiquitin following different treatments of 5-FU, DTN and DHA **(A)** HCT116 cells were treated with single or combination treatment of 5-FU, DTN and DHA for 48h. The protein expression of Mcl-1 was detected using western blotting. **(B)** HCT116 cells were treated with triple combination treatment of 5-FU, DTN and DHA, following the interference of cells by NAC, Spermidine or MG132, ubiquitination of Mcl-1 protein were immunoprecipitated and detected using Western Blot. β-actin was used for loading control. *P <0.05 showed a significant difference compared to control.

Mcl-1 is known to be an unstable protein regulated by degradation. Here, we detected the stabilities of Mcl-1 protein in HCT116 cells by different treatments. As shown in Figure 6A, the lower level of Mcl-1 expression was revealed in HCT116 cells treated with DHA or its combination with 5-FU or DTN compared to other treatment groups, and the significant characteristic of polyubiquitinated Mcl-1 was also detected in HCT116 cells exposed to DHA or its combination with 5-FU or DTN, indicated a close association between Mcl-1 ubiquitination and DHA cytotoxicity (Figure 6B). Interestingly, following the interference of cells by NAC or Spermidine, the elevated effect of polyubiquitinated Mcl-1 were reversed, while interference of MG132 led to the upregulation of Mcl-1 ubiquitination compared to triple combination of DHA+5-FU+DTN, indicated cellular levels of ROS or Spermidine-activated autophagy was associated with the level of Mcl-1 ubiquitination, and the inhibitor of proteasome MG132 can inhibit Mcl-1 ubiquitin degradation induced by triple combination of DHA+5-FU+DTN (Figure 6).

**Figure 7:**
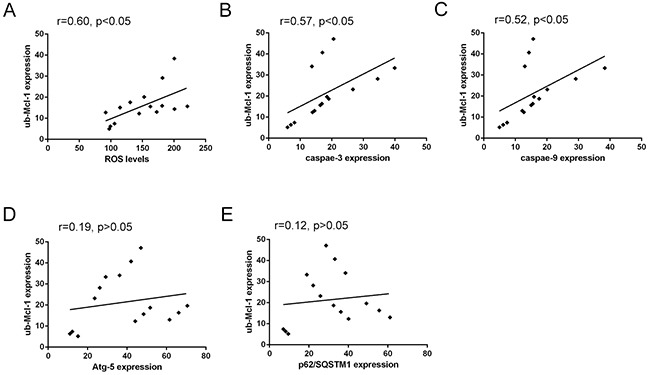
Correlated trend between Mcl-1 polyubiquitination and ROS levels, Atg-5, p62/SQSTM1, Caspase-3 or Caspase-9 expression **(A)** Scatter diagram between Mcl-1 polyubiquitination and ROS levels. **(B)** and **(C)** Scatter diagram between Mcl-1 polyubiquitination and apoptosis-related proteins Caspase-3 and Caspase-9 expression. **(D)** and **(E)** Scatter diagram between Mcl-1 polyubiquitination and autophagy-related proteins Atg-5 and p62/SQSTM1 expression.

In addition, the correlation analysis showed also a close association between the levels of Mcl-1 polyubiquitination and ROS levels, and a moderate correlation with Caspase-3 or Caspase-9, while the correlation between the levels of Mcl-1 polyubiquitination and the expression of Atg5 or p62/SQSTM1 was no significant (Figure 7 and Table 4), indicating the elevation of Mcl-1 polyubiquitination can be enhanced by the elevated ROS levels, and associated with the activation of apoptotic Caspases-3 or Caspase-9, while the change of autophagic-related Atg5 or p62/SQSTM1 may not be a direct factor affecting the characteristic of Mcl-1 ubiquitination. Collectively, results indicated that triple combination of DHA, DTN and 5-FU induces ROS-mediated degradation of Mcl-1 ubiquitination, resulting in the elevated apoptosis in HCT116 cells.

### Effects of 5-FU, DTN, DHA on tumor growth in human colon cancer xenograft in mice

**Figure 8:**
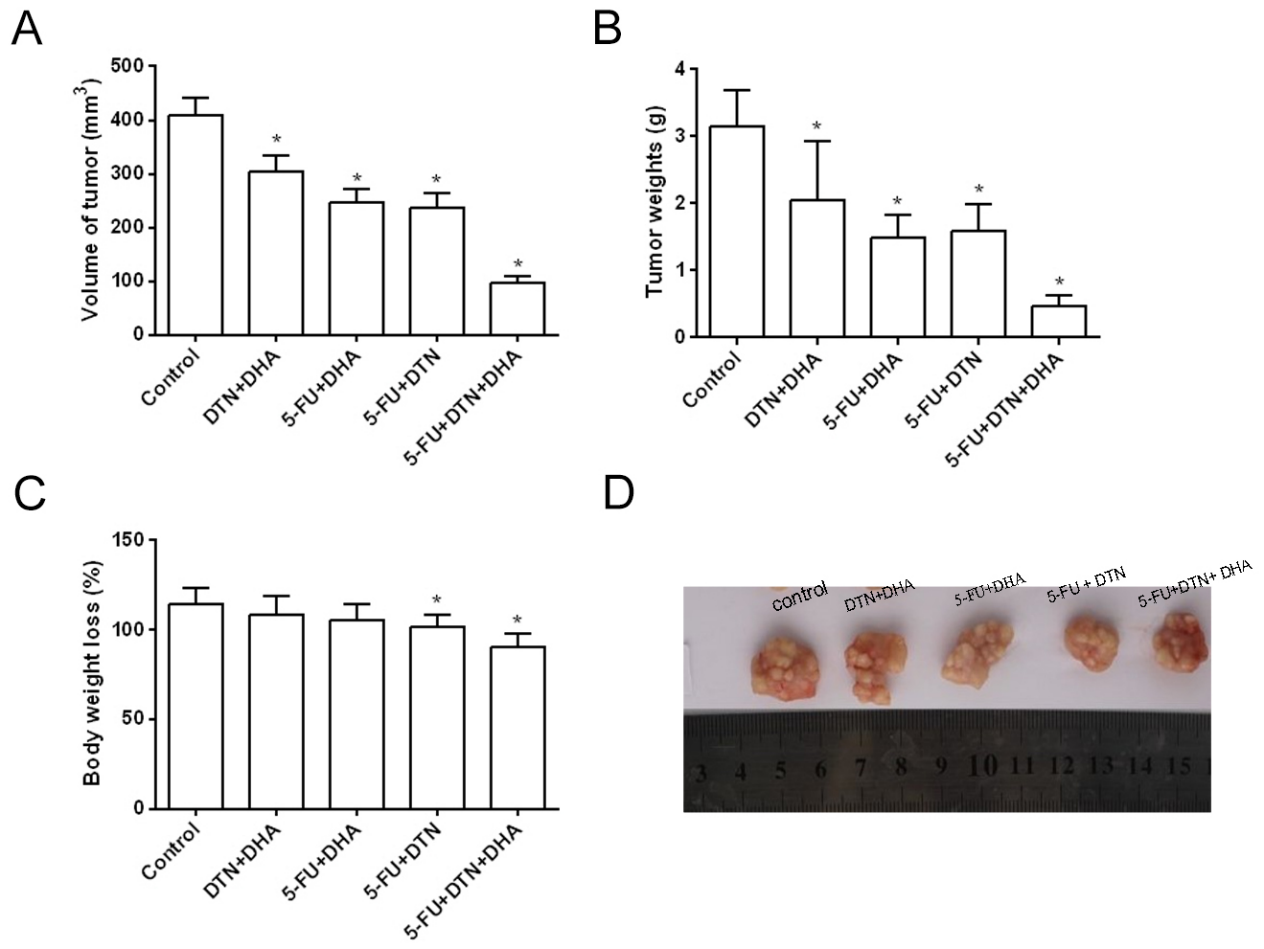
The inhibition effects of colorectal cancer xenografts in mice following combined treatment of 5-FU, DTN and DHA **(A)** and **(B)** The volume of tumor growth and average tumor weights were effectively inhibited after 52 days two or triple combination or 5-FU, DTN and DHA, and the most powerful inhibition occurring in triple combination of 5-FU, DTN and DHA compared to vehicle control. **(C)** The average weight of animals showed a significant loss in the treatment of 5-FU, DTN and DHA compared to day 0. **(D)** Volume of tumors were inhibited by treatment of 5-FU, DTN and DHA. *P <0.05 showed a significant difference compared to control.

**Figure 9:**
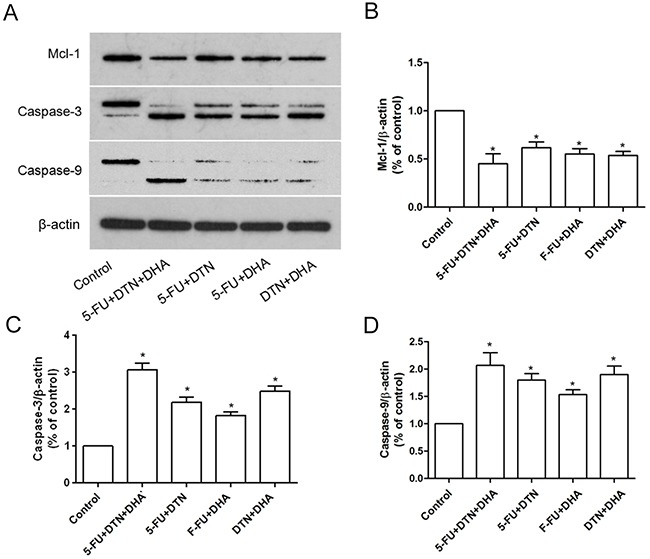
Characteristics of Mcl-1, Caspase-3 and Caspase-9 expression in xenograft tumor mice **(A)** The protein expression of Mcl-1, Caspase-3 and Caspase-9 in xenograft tumor from two or triple combination or 5-FU, DTN and DHA treated mice were detected using western blotting. β-actin was used for loading control. **(B-D)** The densitometric analysis of Mcl-1, Caspase-3 and Caspase-9 expression. *P <0.05 showed a significant difference compared to control.

Results *in vitro* indicated that the triple combination of 5-FU, DTN and DHA showed a significant cytotoxicity in both HCT116 and HCT-8/5-FU cells, which led us to investigate its effect on HCT116 cell xenograft tumor growth in BALB/c nude female mice. Following mice were treated with two or triple combination or 5-FU, DTN and DHA and were observed for 52 days. As compared to the vehicle control group, the triple combination of 5-FU+DTN+DHA significantly reduced the volume and weight of the tumor (*P* < 0.01), and two combinations of 5-FU, DTN and/or DHA showed also a reduction in the volume and weight of the tumor (Figure 8A and 8B). Furthermore, the triple combination of 5-FU+DTN+DHA treatment resulted in 20.9 % loss of body weight (*P* < 0.05) (Figure 8C), as shown in Table 5. Furthermore, the xenograft tumors were excised and processed to Western blotting for determining Mcl-1, Caspase-3 and Caspase-9 protein expression. The Caspase-3 and Caspase-9 protein expression showed a significant elevation in 5-FU+DTN+DHA- treated mice (*P* < 0.01) compared with mice treated with vehicle control (Figure 9). In contrast, there was significant reduction of Mcl-1 expression (*P* < 0.01) in tumors treated with 5-FU+DTN+DHA as compared with vehicle control shown in Figure 9. The significant degradation of Mcl-1 in tumor tissue showed the consistent characteristic with that of Mcl-1 *in vitro* assay. Results suggested the triple combination of 5-FU+DTN+DHA may be an effective strategy for novel colon cancer treatment.

**Table 5:** Volume and weight of tumors, Body weight loss after 52 days treatment of 5-FU, DTN and DHA

	Vehicle control	DTN+DHA	5-FU+DHA	5-FU + DTN	5-FU+DTN+ DHA
Volume of tumor (mm^3^)	410.2±31.5	305.4±28.7*	246.2±24.4*	235.5±27.9*	97.7±10.8*
Tumor weights (g)	3.14±0.55	2.05±0.87*	1.48±0.35*	1.59±0.39*	0.46±0.17*
Body weight loss (%)	114.5±8.7	108.4±10.5	105.8±8.9	101.4±6.8*	90.6±7.5*

Notes: The sample size of BALB/c nude female mice is 40, the number of each group as n=8, respectively; Compared to vehicle control (volume of tumor and tumor weights) or day 0 of treatment (body weight loss), *P <0.05 showed a significant difference.

## DISCUSSION

Colorectal cancer is one of the three most prevalent types of cancers occurring in the worldwide population, including men and women [2, 29]. Due to a lower median overall survival time of only 24 months in colorectal cancer patients with metastatic, the detection and appropriate treatment of early-stage colon cancer is very important in reducing the occurrence and in improving disease prognosis of colorectal cancer victims [30, 31]. In conventional cancer chemotherapy, numerous obstacles exist to prevent successful treatment, such as the poor selectivity of the cytotoxic drugs and the development of multiple drug resistance (MDR) in cancer cells, which represent the most critical challenge to meet. Therefore, the alternative therapeutic strategies are required. Recently, the compound of transitional metal chemistry to target cancer growth pathways and activate cancer cell apoptosis have been developed to decrease the severity of cancer in patients [32, 33]. Among these, thiosemicarbazones such as DpC or TPEN have been found to have potent and selective activity against a range of different tumors [17, 24, 34]. Furthermore, these agents were also demonstrated to overcome chemoresistance through the high affinity for copper, iron and zinc with the higher endogenous levels of ROS in cells [34]. For the unsaturated omega-3 fatty acid, DHA was found to induce several autophagy-related transcripts at early time points in SW620 colon cancer cells whose growth was inhibited by DHA [10]. Furthermore, the activating effect of DHA on UPS system and the pro-apoptotic effect on tumor cells was also confirmed in DHA-treated cells [11, 12].

From a therapeutic viewpoint, the improvement of the anticancer efficacy in cancer formation may be attributed to several fundamental strategies such as high levels of ROS, inhibition of autophagy and activation of UPS system in cells by metal chelators or omega-3 fatty acid. So the combination of 5-FU, DpC, TPEN and DHA is proposed, that might improve the anticancer effects in sensitive or MDR colon cancer cells. In this study, HCT116 and drug resistant HCT-8/5-FU colorectal cancer cells are applied to the treatment in a combination of 5-FU, DTN and DHA, expecting to reveal the anticancer advantage *in vitro* or *in vivo*. Further, the underlying mechanism by the combination of 5-FU, DTN and DHA was also explored to provide a reference for the clinical application. Results showed that two combination of 5-FU, DTN and DHA exhibited an effective cytotoxicity by the decrease of cells viability and increase of ROS levels or apoptosis in HCT116 cells. Interestingly, triple combination of 5-FU+DTN+DHA showed the most powerful cytotoxicity in both HCT116 and HCT-8/5-FU cells by inhibition of autophagy and promotion of apoptosis and ROS generation, while the change of cell viability was blunt in non-cancerous NCM460 cells, indicating the characteristic of selective cytotoxicity and overcome of drug resistance in colorectal cancer cells.

Mcl-1, a well-known Bcl-2 family protein, inhibits apoptosis by binding the proapoptotic proteins such as Bax, Bak or Bim [35]. Here, apoptosis, autophagy and ubiquitination of Mcl-1 protein were detected in (5-FU+DTN+DHA)-treated HCT116 cells, the results showed the increase of ROS levels, polyubiquitination of Mcl-1 protein and apoptosis-related Caspase-3 and Caspase-9 compared to the single treatment or vehicle control groups. Further, following the interference of NAC, Spermidine or MG132, results showed the dynamic change in the levels of Caspase-3, -9 and Mcl-1 ubiquitination which was closely corelated with the change of ROS levels. While, there was no significant correlation between ROS levels and the Atg5 and p62/SQSTM1 expression. The results indicated that the triple combination of 5-FU+DTN+DHA might perform its highly effective cytotoxicity via the pathway of ROS-mediated activation of Mcl-1 ubiquitination and apoptosis-related Caspases enzymes. Moreover, the anticancer effects of the triple combination of 5-FU, DTN and DHA were also demonstrated *in vivo*. Results showed a significant decrease of tumor growth and tumor weight in the triple combination of 5-FU, DTN and DHA. Furthermore, Western-Blotting analysis in the excised xenograft tumors also showed a reduction of Mcl-1 and elevation in the expression of Caspase-3 and Caspase-9 protein.

In summary, the results of *in vitro* and *in vivo* suggest the triple combination of 5-FU+DTN+DHA lead to a significant anticancer selectivity and overcome of drug resistance in colon cancer by the mechanism of ROS-mediated Mcl-1 ubiquitination and apoptosis, may be an effective strategy for novel colon cancer treatment.

## MATERIALS AND METHODS

### Cell culture

Human colorectal cancer cells HCT116 were obtained from the Type Culture Collection of the Chinese Academy of Sciences (Shanghai, China); The multidrug-resistant human colorectal cancer cell line HCT-8/5-FU (MDR) was developed by incubating parental HCT-8 cells (TCCCAS, Shanghai, China) according to the method described by Han et al [36]. As a non-cancerous counterpart, human colonic epithelial NCM460 cells were purchased from INCELL, San Antonio, USA. Cells were maintained in RPMI-1640 medium which were supplemented with 10% (v/v) fetal calf serum, 1% (v/v) nonessential amino acids, 1% (v/v) sodium pyruvate, 12 mM L-glutamine, and 1% (v/v) penicillin/streptomycin (all from Invitrogen). at 37°C in a water-saturated atmosphere with 5% CO_2_, and subcultured by standard methods.

### Reagents

The metal chelator DpC was synthesized as described previously [37]; The acyclic amino metal chelator TPEN was purchased from Sigma-Aldrich, Germany; a representativeomega-3 fatty acid, DHA was supplied by Sigma-Aldrich (Berlin, Germany). Further, the anticancer agent of DpC and TPEN mixture named by DTN was substantially 1:1 diluted in complete RPMI 1640 at 5 mM, respectively, while DHA was dissolved in complete RPMI 1640 at a concentration of 200 μM before experiment [38]. 5-FU, was purchased from Sigma, which was dissolved in DMSO at a concentration of 70 mg/mL and further diluted in filtered water to 13 μg/mL [39]. The final DMSO concentration used on cells is less than 0.3%. Furthermore, for evaluating the role of ROS, autophagy and UPS, the corresponding interfered compound was purchased. In HCT116 cells, ROS scavenger NAC (Beyotime Inc, China) were pretreated by 5 mM for 4 h before scheduled treatment; autophagy inducer Spermidine (Sigma-Aldrich, USA) were treated by 100 μM after scheduled treatment for 6 h; inhibitors of proteasome MG132 (Sigma-Aldrich, USA) were treated by 10 μM after scheduled treatment for 6 h.

### Cell viability assays & drug treatment

Cells viability of HCT116 cells, drug resistant HCT-8/5-FU and noncancerous NCM460 was measured using the MTT assay, according to previously described [40]. Cells were divided into 8 groups of 5-FU, DTN, DHA, 5-FU+DTN, 5-FU+DHA, DTN+DHA, 5-FU+DTN+DHA, and vehicle control. Cells were exposed to anticancer drug 5-FU (5 μg/mL), anticancer agent DTN (DpC: TPEN, 2.5 mM, respectively) or DHA (80 μM), or one combination of 5-FU plus DTN or DHA for 24, 48, 72 h. For further experiment, cells were treated with different treatment for 48 h. The treatment time of medium containing 5-FU or DTN was 48 h, while medium containing DHA was added at the time point of 38 h, and continued to incubate for 10 h [38].

### Apoptosis and autophagic assays

After cells were incubated with experimental agents at 37°C for 48 h, cells were detached, centrifuged and resuspended in 1000 μL binding buffer (10 mM HEPES/NaOH, pH 7.5, 140 mM NaCl and 5 mM CaCl_2_). Cell suspension (5×10^5^) was then incubated with 20 μL Annexin V-FITC reagent (50 μg/ml) and 20 μL PI (50 μg/mL) (Sigma-Aldrich, USA) for 10 min at room temperature in the dark. Then Annexin V-FITC/PI staining positive or negative cells was analyzed by flow cytometry (BD Biosciences, San Jose, CA, USA). For the analysis of autophagy in cells, treated cells were incubated with Acridine orange (AO) solution (5 μg/ml) (Sigma-Aldrich, USA) for 15 min at 37 °C. The media were then carefully discarded and cells were collected in 1× PBS. To eliminate the apoptotic cells, resuspended cells were stained by PI (1 μg/mL) (Sigma-Aldrich, USA) and immediately 10,000 cells per sample was analyzed by flow cytometry (BD Biosciences, USA). The decrease in green fluorescence levels was accepted as autophagy vacuoles through gating cells according to PI-stained untreated cells. Furthermore, the interference of apoptosis or autophagy was performed by NAC or Spermidine or MG132. Then apoptosis and autophagy were assessed as mentioned above.

### Immunoprecipitation and Western blot analysis

For the detection of Mcl-1 ubiquitination, cells were transfected with HA-ubiquitin using Lipofectamine 2000 transfection reagent (Invitrogen, USA) in accordance with the manufacturer's instructions. After 24 h transfection, the cells were then subjected to different treatments for 48 h. Additionally, cells were interfered by NAC or Spermidine or MG132. Treated cells were then lysed in 1% NP-40 buffer (Amresco, USA) and collected for Immunoprecipitation (IP) with anti-Mcl-1 antibody (santa cruz biotechnology inc, USA), following by detection of ubiquitinated Mcl-1 by Western blot analysis. For the method of Western blotting, total cellular protein extract was isolated from the treated cells, the concentration of total proteins was measured by the Bradford method, and proteins were separated by 12% SDS-polyacrylamide gel electrophoresis for further analysis of proteins expression. The primary antibodies used included rabbit polyclonal antibody anti-Caspases 3, anti-Caspases 9 (Bioss Inc, USA), rabbit polyclonal antibody anti-Atg5, anti-p62/SQSTM1 (Novus Biologicals, USA), anti-β-actin rabbit monoclonal antibody Abcam, USA), Following completion of the primary antibody incubating, the membranes were washed several times with TBS/0.1% Tween-20, which was followed by incubation with horseradish peroxidase-conjugated secondary antibodies in room temperature for 1 hour. The membrane was subsequently developed with an enhanced chemiluminescence kit (Walterson Biotechnology, Inc., Beijing, China) and the images were captured and quantified by UVP (Syngene, Frederick, MD, USA).

### Determination of cellular ROS levels

The ROS level was detected by 2′,7′-dichlorodihydrofluorescein diacetate (DCFH-DA) method using OxiSelect™ Intracellular ROS Assay Kit (Cell Biolabs. Inc, USA), according to the manufacturer's instructions. Briefly, cultured cells were treated with different experimental scheme or for 24 h. The cells of 5×10^6^ density were incubated with 10 μmol/L DCFH-DA probes at 37°C for 30 min and washed with PBS 3 times in order to remove the residual probes. Then DCF green fluorescence intensity was detected by flow cytometer (BD Biosciences, USA) at an excitation wavelength of 480 nm and emission wavelength of 530 nm.

### Effect of combination of 5-FU, DTN and DHA in human cancer xenograft model

6–8 weeks old athymic BALB/c nude female mice (Charles River Japan) were subjected to undertake this study. All investigations in animals were carried out after obtaining ethical clearance from Institute animal ethical committee of Xiangya Hospital, Central South University. HCT116 cells were cultured and counted. 5×10^6^ cells were injected subcutaneously in the upper portion of hind legs. Animals were monitored regularly and the development of tumor was monitored using a caliper expressed as length×width×height×0.5236 (mm^3^). When the volume of xenograft tumor reached to 80 mm^3^, these mice were randomly divided into five treatment groups (each group contains 8 mice, Group I: Vehicle control; Group II:5-FU + DTN; Group III: 5-FU+DHA; Group IV: DTN+DHA; Group V: 5-FU+DTN+ DHA). Intratumoral injection schedule of thrice weekly was performed with a dose of 5-FU (500 mg/kg), DTN (DPC: 5 mg/kg; TPEN: 20 mg/kg) and DHA (50 mM), according to the previous protocols [41, 42]. The study was monitored for 52 days by recording animal weight and measuring tumor volume. Subsequently, all animals were sacrificed and tumors were excised for exploring Caspase-3, Caspase-9 protein and Mcl-1 expression.

### Statistical analyses

Data are expressed as the means ± standard deviation by three time repetition in the experiment. The statistical significance of a difference between two groups was analyzed with a two-sided unpaired Student t test, one-way ANOVA and Spearmen correlation analysis was used to analyze the difference and the correlation of apoptosis, ubiquitination and autophagy by SPSS 19.5 software. *P*<0.05 was considered to indicate a statistically significant difference.

## Abbreviations

5-FU: 5-fluorouracil
DpC: di-2-pyridylketone 4-cyclohexyl-4-methyl-3-thiosemicarbazone hydrochloride
TPEN: N, N, N’, N’-tetrakis-[2-pyridylmethyl]-ethylenediamine
DTN: 1:1mixture of DpC and TPEN;
DHA: Docosahexaenoic acid;
MTT: 3-(4,5-cimethylthiazol-2-yl)-2,5-diphenyl tetrazolium bromide
DCFH-DA: 2’,7’-dichlorodihydrofluorescein diacetate
AO: Acridine orange
